# Integrated proteomics and transcriptomics analysis of dynamic changes in muscle fiber types in different regions of porcine skeletal muscle

**DOI:** 10.1007/s44307-025-00080-w

**Published:** 2025-09-24

**Authors:** Zhiting Feng, Xiaoyu Wang, Qingshuang Zhou, Yihao Liu, Rong Xu, Ziyun Liang, Chong Zhang, Xiaohong Liu, Yunxiang Zhao, Yaosheng Chen, Delin Mo

**Affiliations:** 1 State Key Laboratory of Biocontrol, School of Life Sciences, Sun Yat-Sen University, Guangzhou, Guangdong 510275 China; 2 College of Animal Science and Technology, Guangxi University, Nanning, China

**Keywords:** Skeletal muscle, Myogenesis, Mitochondria, Oxidative phosphorylation, Pig

## Abstract

Skeletal muscle serves as a valuable source of nutrition, with distinct muscle fiber types exhibiting different physicochemical properties that influence both meat quality and muscle function. Bama miniature pigs (BM) are recognized for their superior meat quality and their relevance as models for human medical research. Therefore, investigating the differences between slow and fast muscles at various developmental stages (from 57 days post-fertilization to 120 days postnatally) in BM is crucial for both the pork industry and biomedical studies. In this study, we employed a non-targeted data-independent acquisition (nDIA) -based proteomic approach for the first time to porcine embryonic skeletal muscle fibers. A total of 616 differentially expressed genes (DEGs) and 272 differentially abundant proteins (DAPs) were identified in the fast-twitch longissimus dorsi (LD) and slow-twitch semitendinosus (SD) muscles of BM. Domain enrichment analysis and in vitro experiments demonstrated that the NEK3 gene, containing the S_TKc domain, inhibits fast-twitch muscle fiber differentiation postnatally. Additionally, cross-species analysis showed upregulation of skeletal muscle development organ genes in pigs at postnatal day 28. In summary, our results provide both fundamental data and novel insights to further uncover the mechanisms underlying pig skeletal muscle development and muscle fiber transition.

**Supplementary Information:**

The online version contains supplementary material available at 10.1007/s44307-025-00080-w.

## Introduction

Skeletal muscle is crucial for movement and metabolism, accounting for 40% of human body Weight and 75% of total protein (Frontera and Ochala 2015). It also plays a key role in energy homeostasis (Merz and Thurmond 2020; Ceco et al. 2017; Braun and Gautel 2011). In addition to these functions, skeletal muscle is a high-quality source of nutrients and plays a key role in the human diet due to its abundance of essential proteins and amino acids (Hinkle et al. 2022; Liao et al. 2025). However, the nutritional and sensory characteristics of meat derived from skeletal muscle can vary significantly depending on factors such as species, breed, and muscle type (Park et al. 2024). These changes include not only nutritional properties, such as protein content, fat composition, and mineral content, but also extend to sensory qualities, including tenderness, color, and flavor, all of which influence consumer preferences (Park et al. 2024). These differences are largely attributed to the composition of muscle fibers, as they are the fundamental structural components of muscle tissue and directly affect the texture, juiciness and sensory characteristics of meat (Ryu et al. 2005; Joo et al. 2013; Park et al. 2022). Research shows that the specific ratio of slow muscle fibers to fast muscle fibers, as well as the metabolic pathways they utilize, have a profound impact on the texture and quality of meat. For instance, meat produced by fast-contracting fibers is tougher and has higher glycolytic activity, while meat made from slow-contracting fibers is more tender and has a higher oxidation capacity (Xu et al. 2020).

Skeletal muscle development is a complex, dynamic process involving several stages, from the formation of muscle fibers in embryogenesis to tissue maturation and remodeling postnatally (Yang et al. 2021a, b). Myogenesis, which involves the differentiation of myoblasts into muscle fibers, occurs during the prenatal and postnatal stages. Prenatal myogenesis comprises primary and secondary phases: primary myogenesis, driven by embryonic myofibroblasts, occurs early in development, while secondary myogenesis, involving fetal myofibroblasts, takes place later in gestation, between 55 and 90 days in pigs (Wang et al. 2021). These processes establish the muscle fiber pool, which undergoes maturation and adaptation postnatally. Postnatal remodeling involves fiber size changes and type conversion (e.g., slow- to fast-twitch fibers) (Braun and Gautel 2011), regulated by signaling pathways such as calcineurin (Swoap et al. 2000) and calmodulin kinase (McKinsey et al. 2000; Grondard et al. 2008),alongside regulators of mitochondrial activity and metabolism (Voillet et al. 2018). However, the molecular mechanisms, particularly during postnatal development, are poorly understood (Lamon et al. 2017; Wang et al. 2021). To address this, we applied advanced proteomic techniques (nDIA/MS–MS), offering improved speed and sensitivity over conventional LC–MS/MS (Guzman et al. 2024), to examine protein changes during muscle fiber transitions.

The Bama miniature pig (BM) is a unique breed in Bama Yao Autonomous County, Guangxi, China. Due to its excellent meat quality, adaptability to various environmental conditions and tolerance to roughage, it is an ideal model for muscle development research (Pan et al. 2021). Furthermore, its small size and ability to thrive under various conditions make it an important contributor to local economic development and agricultural growth, especially in poverty alleviation efforts (Zhu et al. 2018; Zhu et al. 2020a, b). The BM has also been valued for its early sexual maturity, high litter size and high degree of inbreeding, and has become an important animal model in genetic research (Yang et al. 2018). And the BM pigs also be used as medical disease models (Zhu et al. 2018). Despite these advantages, this breed also has some limitations, including a low lean meat ratio, high fat content, slow growth rate, and poor feed conversion efficiency (Zhu et al. 2018; Zhu et al. 2020a, b). To explore the molecular basis of muscle development in this breed, we collected longissimus dorsi (LD) and semitendinosus (SD) samples at six developmental stages: days 57, 73, and 90 of gestation, and 1, 28, and 120 days after birth. LD is predominantly composed of glycolytic type ⅡB (fast-twitch) fibers, while SD contains more oxidative type Ⅰ (slow-twitch) fibers (Wang et al. 2020). This sampling enables the identification of key time points for muscle fiber transformation and provides insights into the dynamics of gene expression related to growth, meat quality and feed efficiency. In addition, understanding the changes in gene expression over time can help identify proteins that improve meat quality, growth rate and feed efficiency. Moreover, due to its small size and genetic stability, BM is frequently used in disease modeling (Zhu et al. 2020a, b). Thus, this study not only contributes to the improvement of local pig breeds, but also provides a deeper understanding of muscle biology that can inform strategies to enhance muscle health and disease management in humans. Ultimately, this study will provide valuable insights into the genetic potential of local pig breeds, enhance their economic value, and advance scientific and agricultural knowledge.

## Materials and methods

### Animals and samples preparation

The longissimus dorsi (LD) and semitendinosus (SD) muscle samples were collected from fetal and early postnatal BM pigs, all resulting from natural conception. The tissue samples of the offspring at 57, 73, and 90 days after fertilization and 1, 28, and 120 days after birth were labeled as E57, E73, E90, P1, P28, and P120 respectively. The fetuses (3 at each stage) are delivered by cesarean section, while the pigs (3 at each stage) are born naturally.

### Histology

The LD and SD samples from 18 pigs across six developmental stages were preserved in 4% paraformaldehyde, embedded in paraffin, sliced into 6-μm-thick sections, and stained with hematoxylin for 10 min, followed by 70% ethanol and eosin for 5 min. The samples were then dehydrated with alcohol and cleared using xylene. The samples were examined and photographed under a microscope (Nikon, Japan).

### RNA sequencing (RNA-seq)

Total RNA was extracted using TRIzol (Genstar, Beijing, China) according to the manufacturer's instructions. RNA concentration was determined using a NanoDrop 3000 (Thermo Fisher, Waltham, MA, USA), and samples were stored at − 80 °C. Transcriptome libraries were prepared for sequencing using the KAPA Stranded RNA-Seq Library Prep Kit (Illumina). Sample clustering was conducted using KAPA RNA Adapters set1/set2 for Illumina. After clustering, the libraries were sequenced using 2 × 150 bp paired-end modules on the Illumina NovaSeq 6000 platform.

### Proteomics analysis

Tissue samples were pulverized in liquid nitrogen and placed into 5 mL centrifuge tubes, then kept at − 80 °C. Prior to use, the samples were retrieved, ground while cold, and promptly transferred to tubes pre-cooled with liquid nitrogen. SDT buffer (with 100 mM NaCl) and DTT (1/100 volume) were added, followed by vortexing and ultrasonic lysis in an ice-water bath for 5 min. Then heat the lysis buffer to 95° C for 8–15 min, cool it on ice for 2 min, and centrifuge it at 12,000 g at 4° C for 15 min. Collect the supernatant, treat it with iodoacetamide (IAM), and incubate in the dark for 1 h. Add 4 volumes of pre-cooled (−20 °C) acetone to precipitate the protein, incubate at −20 °C for at least 2 h, and centrifuge under the same conditions. The obtained particles, representing the total protein, are dissolved in an appropriate volume of DB buffer to ensure complete dissolution.

For digestion, the protein solution was adjusted to 100 μL with DB buffer (8 M urea, 100 mM TEAB, pH 8.5), followed by the addition of trypsin and TEAB. After incubation at 37 °C for 4 h, more trypsin and CaCl₂ were added for overnight digestion. The pH was adjusted to < 3 with formic acid. After centrifugation at 12,000 × g for 5 min at room temperature, the supernatant was desalted on a C18 column, washed three times in 3% acetonitrile with 0.1% formic acid, and eluted in 70% acetonitrile with 0.1% formic acid. The eluate was collected and lyophilized. Liquid chromatography-mass spectrometry was used to prepare mobile phase A (100% water-0.1% formic acid) and mobile phase B (80% acetonitril-0.1% formic acid). The dried peptides were dissolved in 10 μL of mobile phase A, centrifuged at 14,000 × g for 20 min at 4° C, and 200 ng of the supernatant was injected for mass spectrometry. Chromatographic separations were performed on a Vanquish Neo nano-UHPLC system. A C18 trap column (5 mm × 300 μm, 5 μm, Thermo, part no. 174500) was used, and samples were eluted through a C18 analytical column (PepMap™ Neo UHPLC, 150 μm × 15 cm, 2 μm, Thermo) maintained at 50 °C. Detection was conducted on a Thermo Orbitrap Astral mass spectrometer with an Easy-Spray ESI source (1.9 kV, 290 °C). Data-independent acquisition (DIA) mode was used with a full MS scan range of m/z 380–980, 240,000 resolution at m/z 200, AGC target of 500%, 2 Th isolation window, 300 DIA windows, The normalized collision energy (NCE) of 25%, and MS/MS scan range of m/z 150–2000 at 80,000 resolution and 3 ms maximum injection time. Raw data (.raw) were acquired.

Protein identification and quantification were performed using DIA-NN against a reference protein database. Parameters included a 10 ppm precursor mass tolerance and 0.02 Da fragment ion tolerance. Carbamidomethylation of cysteine was set as a fixed modification; variable modifications included methionine oxidation, N-terminal acetylation, methionine loss, and methionine loss plus acetylation. One missed cleavage was allowed. Peptide-spectrum matches (PSMs) with ≥ 99% confidence was retained, and results were filtered to exclude peptides and proteins with a false discovery rate (FDR) > 1%.

### Proteome and transcriptome analysis

All downstream analyses and statistical computations were conducted using R/Bioconductor. Principal component analysis (PCA) was performed using the "prcomp" function in R. Functional enrichment analysis was carried out with the "enricher" function from the clusterProfile (Yu et al. 2012) package. Proteins with a fold change (FC) ≥ 1.2 or ≤ 0.833 and a p-value < 0.05 were defined as region-specific DAPs. In contrast, stage-specific DAPs were identified based on an adjusted p-value (FDR) < 0.05. For transcriptomic data, genes with |log₂FC|> 1 and FDR-adjusted p < 0.05 were considered differentially expressed in stage comparisons, while region-specific DEGs used the same |log₂FC| threshold but unadjusted p-values. This difference in statistical criteria reflects the relatively subtle variation between regions, where FDR correction would substantially reduce the number of detected DAPs and DEGs. Therefore, a more lenient threshold was applied in regional comparisons. All heatmaps were generated using the pheatmap package in R. Pearson correlation coefficients between stages were computed using the"cor"functions in R. The upset plot was generated using the UpSetR (Conway et al. 2017) package in R. Time-dependent expression patterns for each region were analyzed using the Mfuzz (Kumar and Futschik 2007) package. Additionally, we compared the transcriptomic data from the published study (Yang, et al. 2021a, b) with our own proteomic and transcriptomic expression data. To ensure compatibility across datasets and minimize technical variation, we applied the ComBat() function from the SVA package in R to correct for batch effects. This approach accounted for differences in sequencing batches while retaining key biological variability by including pig breed and sampling region as fixed effects. Following batch correction, all datasets were standardized using Z-score normalization. The resulting harmonized data were then integrated for direct comparative analysis.

### Protein domain analysis

Domain information for all proteins was retrieved from the SMART (Letunic et al. 2021) database (https://smart.embl.de). A schematic of the NEK3 protein domain was derived from predictions made by AlphaFold3 (Abramson et al. 2024).

### Ortholog gene selection

Orthologues of genes encoding muscle proteins were first retrieved from the Ensembl database. To identify cross-species orthologues, we performed species comparison using OrthoFinder (Emms and Kelly 2019), incorporating FASTA files from Homo sapiens (updated on 2025-02-02) and Mus musculus (updated on 2025-01-30). Following the OrthoFinder analysis, we selected sequences categorized as Single_Copy_Orthologue_Sequences to ensure high-confidence homology. These protein sequences were then mapped back to their corresponding coding genes using Ensembl’s annotation tools. As a result, a total of 1,869 protein-coding genes were identified across the three species. To explore their expression dynamics, transcriptomic data for human and mouse skeletal muscles were obtained from publicly available datasets, GSE257558 for humans and GSE167665 for mice. Expression trends of the identified orthologous genes were visualized using the ClusterGVis package, allowing cross-species developmental comparisons. For humans (Schaiter et al. 2024) (*Homo sapiens*, Germany), samples from postnatal development at 7 months were collected from the quadriceps femoris. In mice (Izgi et al. 2022) (*Mus musculus*, outbred strain C57Bl/6J), samples were collected at postnatal day 20 (P20) from skeletal muscle.

### Cell culture

Embryonic muscle progenitors were extracted from the LD of BM 35 days post-coitum. The isolated LD was cleaned of connective tissues, chopped, and digested with 0.2% collagenase type I (Sigma, Shanghai, China) solution in a water bath at 37 °C for 2 h to obtain sufficient cells. Cells were cultured in Dulbecco’s modified Eagle medium (DMEM; GIBCO, New York, USA) with 20% (v/v) fetal bovine serum (FBS; GIBCO), 1% penicillin–streptomycin antibiotics (MesGenBiotech, Shanghai, China), and 0.5% chicken essential extract (growth medium, GM).

C2C12 cells were sourced from the American Type Culture Collection and cultured at a subconfluent density in Dulbecco’s Modified Eagle Medium (DMEM; Corning, USA) with 10% fetal bovine serum and 1% penicillin/streptomycin (growth medium).

To induce differentiation, the cells were transferred to DMEM with 2% horse serum (Gibco, USA) and 1% penicillin/streptomycin once they reached confluence. All cultures were maintained in a humidified incubator at 37 °C with 5% CO2.

### Immunohistochemistry

The freshly isolated tissues were fixed in 4% paraformaldehyde for 24 h, then dehydrated in a graded ethanol series and embedded in paraffin. 5 μm thick sections were cut from the paraffin block using a rotary slicer (Microm HM 340 E, Germany). Antigen remediation was performed with sodium citrate and then permeated in 0.5% Triton X-100 for 15 min. After blocking with 5% bovine serum albumin (BSA) for 30 min, incubate overnight with the primary antibody. The next day, the sections were incubated with the corresponding fluorescent secondary antibody for 1 h, re-stained with DAPI, and imaged under a fluorescence microscope (Leica TCS-SP8 from Germany). Detailed antibody information can be found in Supplementary Table 8. The fusion index is the ratio of the number of nuclei with more than three nuclei in the intramuscular canal to the total number of nuclei.

### Western blotting

The cells were washed with cold phosphate-buffered saline (PBS) and lysed with radioimmunoprecipitation assay buffer (Applygen Technologies, Beijing, China) containing 1% protease inhibitor (Roche, Basel, Switzerland). Approximately 20 μg of protein per lane was separated by sodium dodecyl sulfate polyacrylamide gel electrophoresis and transferred to a polyvinylidene difluoride membrane at 250 mA for 2 h. The membranes were blocked with 5% skim milk for 2 h and then incubated overnight at 4 °C with the corresponding primary antibodies (Supplementary Table 8). Subsequently, the membranes were washed with Tris-buffered saline containing Tween-20 and incubated with horseradish peroxidase-conjugated anti-rabbit IgG or anti-mouse IgG secondary antibodies. Finally, the bands corresponding to the target proteins were visualized using an enhanced chemiluminescent substrate (Millipore, Billerica, USA) and observed using a Gel Doc XR System (Bio-Rad). Band densities were analyzed using Image Lab software (Bio-Rad). Detailed information on the antibodies used is provided in Supplementary Table 8.

### RNA extraction and quantitative real-time PCR (RT-qPCR)

Total RNA was extracted from cells using TRIzol reagent (Genstar, Beijing, China), and the RNA's quantity and quality were evaluated with a NanoDrop 3000 spectrophotometer (Thermo Fisher, Waltham, USA). Subsequently, total RNA was reverse transcribed into cDNA using a Primer-Script RT Reagent Kit (Genstar, Beijing, China), employing a random primer for the protein-coding genes assay. RT-qPCR was then conducted using a One-Step SYBY PrimeScript RT-PCR kit (Genstar, Beijing, China) on an Applied Biosystems QuantStudio 7 Flex Real-Time PCR System (ABI, USA). β-actin served as the internal reference gene to normalize the expression levels of protein-coding genes and microRNAs. Finally, the 2^−ΔΔCt^ method was used to calculate the relative fold changes in the expression of the genes of interest. Detailed primer information is provided in Supplementary Table 8.

### Statistical analysis

Fusion index was shown as mean ± SD. Two tailed student’s t-test was used to compare the differences between the groups, and the threshold of significance was set at *p* < 0.05. The p-value of DEGs, DAPs, GO term and KEGG pathway enrichment were adjusted by the false discovery rate (FDR) method.

## Results

### Proteome of Muscle Development in Bama Miniature pigs

**Fig. 1:**
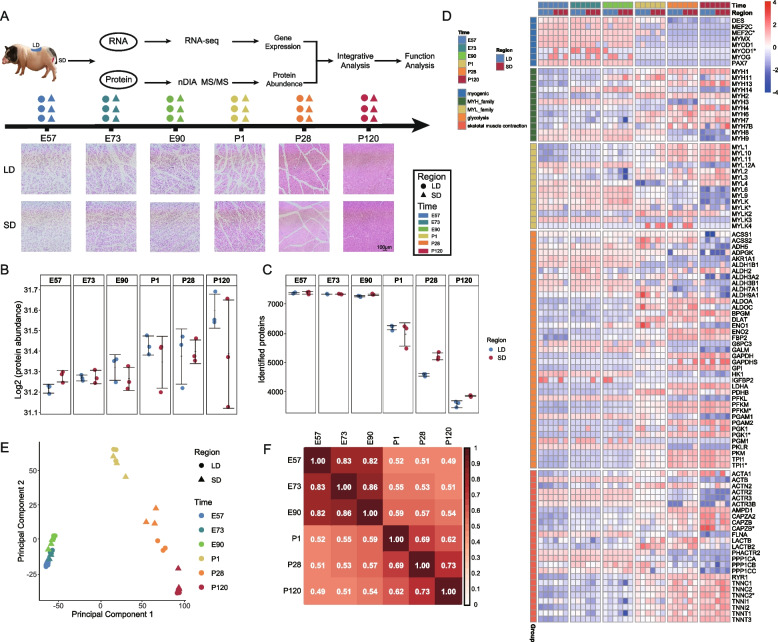
Sampling overview and global proteomic profiles across skeletal muscle development in Bama miniature pigs. **A** A schematic representation of skeletal muscle regions and the developmental time points at which samples were collected from Bama miniature pig muscle. A total of two muscle regions (LD and SD) at six developmental stages (E57, E73, E90, P1, P28, P120) were profiled using proteomic analysis. The specific sampling regions at each stage are color-coded in the upward panel. Each region is represented by three biological replicates from distinct animals at the indicated stages. The downward panel displays hematoxylin and eosin (HE) staining results of skeletal muscle sections from different regions at various time points. **B**, **C** The total protein abundance (**B**) and the number of identified proteins (**C**) in the two regions at the six stages, with three biological replicates per stage, are shown. The average number of identified proteins per stage is presented. The colors corresponding to each region are consistent with those in panel (**A**). The dots, horizontal lines, and error bars represent individual sample values, mean values, and standard deviations, respectively. **D** Heatmap depicting the abundance patterns of key proteins categorized into five groups: myogenic genes, MYH family, MYL family, glycolysis-related genes, and skeletal muscle contraction-related genes (*n* = 3 per group). **E** PCA displaying the distribution of all samples based on protein abundance, with samples grouped by stage. LD samples are denoted by circles, while SD samples are represented by triangles. **F** Correlations between the six stages based on protein abundance

The natural gestation period of BM pigs is approximately 114 days. Six key developmental time points were selected for this study: embryonic days 57, 73, and 90 (E57, E73, and E90), and postnatal days 1, 28, and 120 (P1, P28, P120). These time points correspond to critical stages of secondary muscle fiber development and type transitions (Wigmore and Stickland 1983). Skeletal muscle samples were systematically dissected from 18 pigs (three biological replicates per stage) to isolate two distinct muscle regions, the LD and SD, which are associated with fast-twitch and slow-twitch fibers, respectively (Fig. 1A). The protein profiles of these 36 samples were quantified using the nDIA proteomics strategy. Protein abundance was expressed as Label-free quantification (LFQ) intensity and transformed log2 prior to differential expression analysis (Fig. 1B and Supplementary Table 1). On average 6118 proteins were identified per sample (range from 3468 to 7416 proteins) (Fig. 1C and Supplementary Table 2). The highest and lowest average number of proteins were identified at E57 and P120, respectively (Fig. 1C). A protein abundance heatmap (Fig. S1A) was generated, which revealed three distinct expression patterns: a postnatal decrease, a postnatal increase, and a significant upregulation starting at postnatal day 28. Additionally, we constructed heatmaps for key genes involved in myogenesis, the MYH and MYL families, glycolysis, and skeletal muscle contraction (Fig. 1D). While regional differences were minimal, gene expression patterns exhibited two major trends across developmental stages: a decline or increase around birth. This suggests that these key genes play a regulatory role in muscle development. PCA of skeletal muscle regions revealed that, although samples from prenatal stages clustered relatively closely, a subtle separation according to developmental stage could still be observed. In contrast, postnatal samples exhibited clear and distinct separation across different time points (Fig. 1E). Samples from earlier stages were distinctly separated from those at later stages, with the fetal stages (E57-E90) exhibiting high similarity and being tightly clustered. Correlation analysis demonstrated significant correlations between adjacent stages, with the highest correlation (0.86) observed between E73 and E90 (Fig. 1F). Correlations were notably high between adjacent stages in both the pre- and postnatal periods (0.82–0.86 and 0.62–0.73, respectively), although the correlation between E90 and P1 was only 0.59, further supporting the clustering results from PCA.

### Dynamic Proteomic Changes Across Developmental Stages and Muscle Regions

**Fig. 2:**
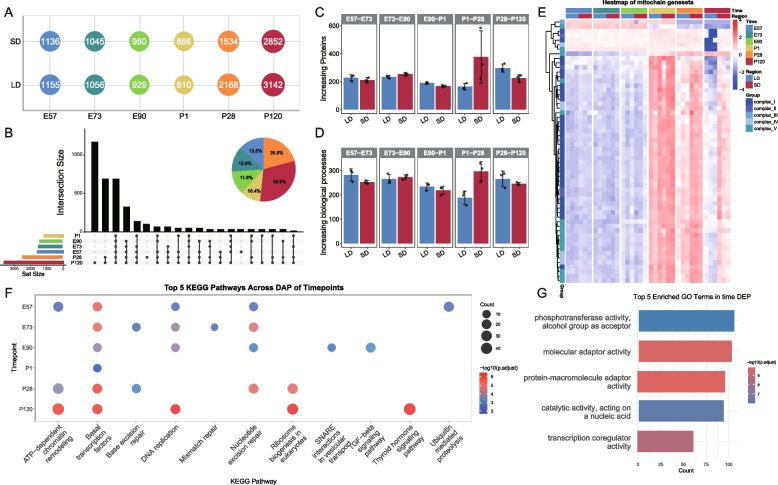
Significant differences between developmental stages and muscle regions are revealed by spatiotemporal proteomics. **A** The number of DAPs across different muscle regions and developmental stages is presented. The DAPs for each region were identified by comparing them to other muscle regions at the same developmental stage. **B** An UpSet plot illustrating the number of DAPs and their overlaps at each stage group. The accompanying pie charts show the proportion of DAPs within all identified DAPs during the respective period. **C**, **D** The number of increasing proteins (**C**) and related biological processes (**D**) in the LD and SD regions when comparing adjacent stages. **E** A heatmap depicting differentially abundant proteins associated with respiratory complexes in skeletal muscle (*n* = 3 for each group). **F** Comparison of the top 5 KEGG biological processes that were enriched at different developmental stages. **H** The top 5 GO biological processes that became enriched during different stages of DAPs

DAPs were identified using the Student’s t-test, followed by FDR for multiple testing. The number of DAPs across the six developmental stages and two regions is presented in Fig. 2A. A total of 3,509 DAPs were identified across developmental stages, exceeding the number identified between regions (272 DAPs). All developmental DAPs were visualized in an upset plot highlighting the top 20 pairwise comparisons to maximize gene inclusion (Fig. 2B). It is worth noting that the pie chart shows that P120 has the highest number of DAP (30.9%), followed by P28 (20.8%). The overall proteomic profiles of different skeletal muscle regions did not exhibit clear separation across developmental stages in the PCA plot (Fig. 1E). To explore this further, we conducted pairwise comparisons between adjacent stages to quantify proteins with increased expression and newly enriched biological processes (Fig. 2C, D). Proteins classified as"increasing” were those that appeared at a given stage (LFQ intensity > 0) but were absent in the preceding stage (LFQ intensity = 0). Between P1 and P28, the SD region showed more newly expressed proteins than the LD region. We also determined the"decreasing proteins"by subtracting the expression level of the preceding stage from the expression level of the given stage (Fig. S1B, C). Similar to the trend observed in increasing proteins, significant differences in both protein abundance and enriched biological processes were detected between the two muscle regions during the P1-P28 transition. This suggests that the P1-P28 period may be a critical phase for muscle fiber transformation.

During skeletal muscle development, previous studies suggest that mitochondrial gene expression may drive muscle fiber transitions (Wang et al. 2024). To explore this, we generated a heatmap of DAPs (p < 0.05) associated with mitochondrial complexes across the two muscle regions at each developmental stage (Fig. 2E). From P28 onward, a significant divergence in the abundance of mitochondrial complex-related proteins between the muscle regions was observed, with the most pronounced differences occurring at P120. The emergence of mitochondrial protein expression differences at P28 suggests this stage is a critical period for muscle fiber type transitions. This finding aligns with the results observed in the analysis of increasing and decreasing proteins. KEGG enrichment analysis of proteins with significant abundance differences of developmental stages primarily identified pathways associated with the basal transcription factors and nucleotide excision repair (Fig. 2F), while GO enrichment analysis highlighted molecular adaptor activity as key enriched processes (Fig. 2G).

**Fig. 3:**
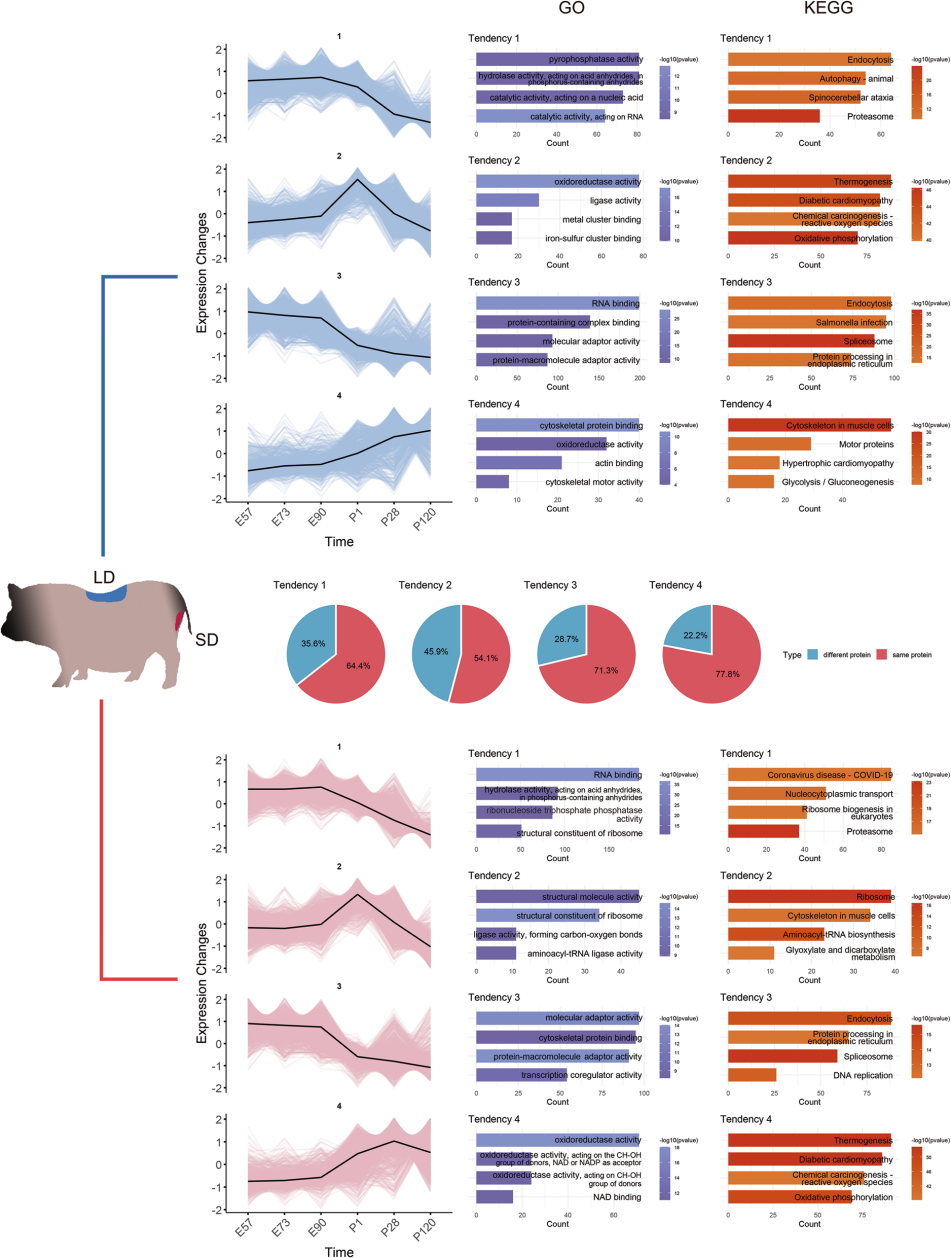
Main biological processes involved in skeletal muscle growth. The distribution of expression for each region group and the four most significant items for each trend (Tendency 1–Tendency 4) within the two groups is presented. The x-axis represents time points, while the y-axis represents the density of the data points in the line plot. The black line indicates the overall expression levels for each trend. The middle panel presents the proportion of shared and distinct proteins within the same tendency across the two muscle regions

Although consecutive comparisons across various developmental stages and muscle regions provide valuable insights into muscle growth, the enrichment results did not yield direct evidence regarding the specific processes underlying muscle development. To further investigate the expression patterns of DAPs and their involvement in postnatal skeletal muscle growth, all proteins were categorized by muscle region (LD and SD) and further classified into four distinct trends (Tendency 1–Tendency 4, Fig. 3) based on their abundance patterns. Although overall protein abundance did not significantly differ between LD and SD, the two regions exhibited differences in protein composition and enriched pathways (Fig. 3). For each trend, we calculated the proportion of shared and unique proteins between LD and SD, finding that 22.2%–45.9% of proteins differed. This indicates that despite similar abundance trends, protein composition and associated pathways varied considerably between regions. Functional enrichment analysis revealed that both muscle regions shared similar but distinct biological pathways, with four representative terms selected from GO and KEGG analyses (Fig. 3) and the top 10 significantly enriched pathways for each trend (Fig. S2–5).

Both LD’s and SD’s Tendency 1 exhibited a gradual decline in expression from E90. Both regions were associated with hydrolase activity and proteasomal pathways, but LD also showed enrichment in catalytic activity, endocytosis, and autophagy, while SD was enriched in nucleocytoplasmic transport and ribosome-related pathways. In Tendency 2, both regions peaked around the birth period. KEGG enrichment for both showed a focus on ligase activity, though LD was enriched in thermogenesis-related processes, while SD was more associated with ribosome-related pathways, indicating distinct functional roles at birth. Tendency 3 showed a sharp decline in protein abundance around birth, with pathways related to molecular adaptor activity, endocytosis, spliceosome function, and protein processing in the endoplasmic reticulum. These processes are crucial for muscle development and suggest a shift in muscle function during this stage. Finally, tendency 4 in both regions showed similar increasing abundance patterns over time, suggesting a role in metabolic regulation. Both regions exhibited enrichment in oxidoreductase activity-related pathways. Interestingly, LD was enriched in glycolysis and gluconeogenesis, while SD showed significant enrichment in oxidative phosphorylation, reflecting the different energy metabolism characteristics of fast and slow muscle fibers. These findings suggest that different biological pathways regulate fast and slow muscle development, shaping the fiber composition of each muscle type.

Genes exhibiting expression trends of potential biological significance were selected and compared with publicly available transcriptomic data (Yang et al. 2021a, b). This comparison revealed that our data closely align with published transcriptomic datasets, demonstrating reproducibility (Fig. S6). Of note, published transcriptomic data showed more similarity to our proteomic data than to our transcriptomic data, highlighting the importance of multi-omics comparisons.

### Differential Protein Domain Involvement in Muscle Fiber Transition Regulation Between Muscle Regions

**Fig. 4:**
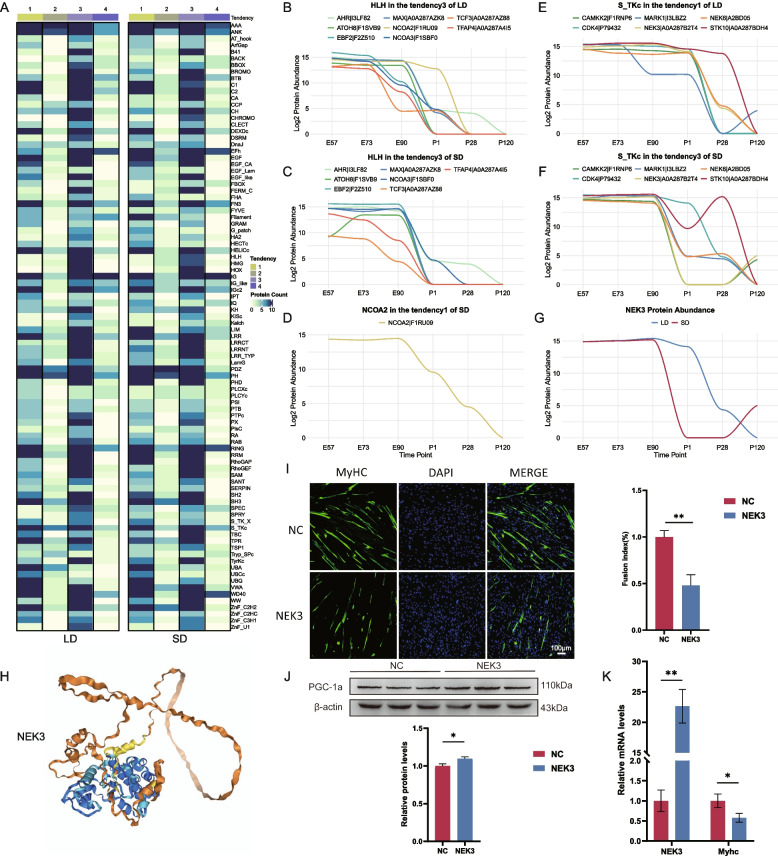
Protein domain distribution and abundance patterns in skeletal muscle regions. **A** Distribution of protein domains across each tendency in skeletal muscle regions. **B** Abundance patterns of HLH domain-associated proteins in Tendency 3 in the LD. **C** Abundance patterns of HLH domain-associated proteins in Tendency 3 in the SD. **D** Abundance patterns of NCOA2 in Tendency 1 in the SD. **E** Abundance patterns of S_TKc domain-associated proteins in Tendency 4 in the LD. **F** Abundance patterns of S_TKc domain-associated proteins in Tendency 3 in the SD. **G** Abundance patterns of NEK3 in LD and SD. **H** Three-dimensional structures of NEK3, predicted by AlphaFold3. The interface predicted template modeling (ipTM) score = 0.58. **I** MyHC immunofluorescence staining after six days of myogenic induction. Upper panel: MyHC (green); DAPI (blue). Scale bar: 100 μm (*n* = 3). Lower panel: Fusion index of cells. **J** Western blot analysis of PGC-1α expression in NC and NEK3-overexpressing porcine primary cells (*n* = 3). **K** Quantification of NEK3 and MyHC mRNA levels in NC and NEK3-overexpressing porcine primary cells (*n* = 3). *P < 0.05, **P < 0.01, **P < 0.001 (Student’s t-test)

Protein–protein interactions and the biological functions of proteins are often mediated by their structural domains. We analyzed the distribution of protein domains across abundance trends (Fig. 4A) and individual proteins (Supplementary Table 3). Enrichment analysis of the domains revealed that Trend 3 was consistently enriched in helix loop helix domain (HLH) domains, while Trend 1 in LD and SD—both showing a continuous decline from E90 onward—shared similar domain enrichment patterns with Trend 3. However, each trend also exhibited unique domain enrichments, such as ubiquitin associated domain (UBA) in LD tendency 2, and significant enrichment in filaments in SD tendency 2 and LD tendency 4 (Fig. 4A).

Although the HLH domain showed minimal differences between the two muscle regions, its relevance to muscle fiber transition (Chen et al. 2000) prompted us to further investigate the changes in protein abundance for HLH-containing proteins between LD and SD. The analysis demonstrated that the majority of proteins exhibited similar abundance patterns (Fig. 4B, C), with the exception of Nuclear Receptor Coactivator 2 (NCOA2), which displayed distinct expression trends. As illustrated in Fig. 4B, NCOA2 followed a pattern akin to Trend 3, characterized by a sharp postnatal decline in abundance (Fig. 4A). However, in the SD region, NCOA2 adhered to Trend 1, with a gradual reduction beginning at E90 (Fig. 4D). Differences in abundance regulation between muscle regions may contribute to differences in fast and slow fiber composition between the two skeletal muscle types. NCOA2 enhances the differentiation of cultured C2C12 myoblasts into myotubes by enhancing myocyte enhancer factor (MEF)−2C transactivation (Chen et al. 2000), although it is a myogenic differentiation 1 (MyoD) -mediated transcriptional co-repressor (Wu et al. 2005). These findings highlight the crucial role of NCOA2 in muscle development. The temporal and spatial differences in its abundance across skeletal muscle types may influence muscle fiber transitions, ultimately contributing to variations in fast-twitch and slow-twitch fiber composition between the two regions.

Notably, while between two skeletal muscles both Trend 1 and Trend 3 were associated with the S_TKc domain (Fig. 4A), the corresponding proteins showed region-specific abundance patterns (Fig. 4E-G, Fig. S7A). We observed that the protein compositions in LD Trend 1 and SD Trend 3 were almost identical but exhibited different temporal patterns, suggesting that these regional domains may play different roles in regulating myofiber type transitions. To investigate the potential function of NEK3, we used AlphaFold3 to predict its structural model (Fig. 4H) and performed in vitro functional validation. C2C12 cells were transfected with NEK3 or negative control (NC). NEK3 overexpression significantly inhibited MYHC expression (Fig. S7B), a result further supported by RT-qPCR analysis (Fig. S7C), confirming a significant reduction in MYHC transcript levels. Furthermore, western blot analysis revealed that overexpression of NEK3 inhibited the expression of fast muscle fiber markers MYH4, while upregulating slow muscle fiber markers MYH7 and PGC-1α (Fig. S7D), indicating that NEK3 negatively regulates the differentiation of fast muscle fibers. To verify these findings in more physiologically relevant models, we conducted a functional analysis of embryonic muscle progenitor cells. The overexpression of NEK3 significantly inhibited the myogenic differentiation of embryonic muscle progenitor cells, which can be demonstrated by the reduction of myotube formation (Fig. 4I, K). Western blot analysis further confirmed that NEK3 inhibited the expression of rapid twitching specific markers in embryonic muscle progenitor cells (Fig. 4J), which was consistent with the observations in mouse C2C12 cells. These results indicate that NEK3 has a conserved inhibitory effect on the differentiation of fast-twitch muscle fibers across species. Overall, these findings suggest that NEK3 plays a negative regulatory role in muscle formation and the norms of fast-contracting muscle fibers.

### Dynamic transcriptomic changes and discrepancies between proteome and transcriptome

**Fig. 5:**
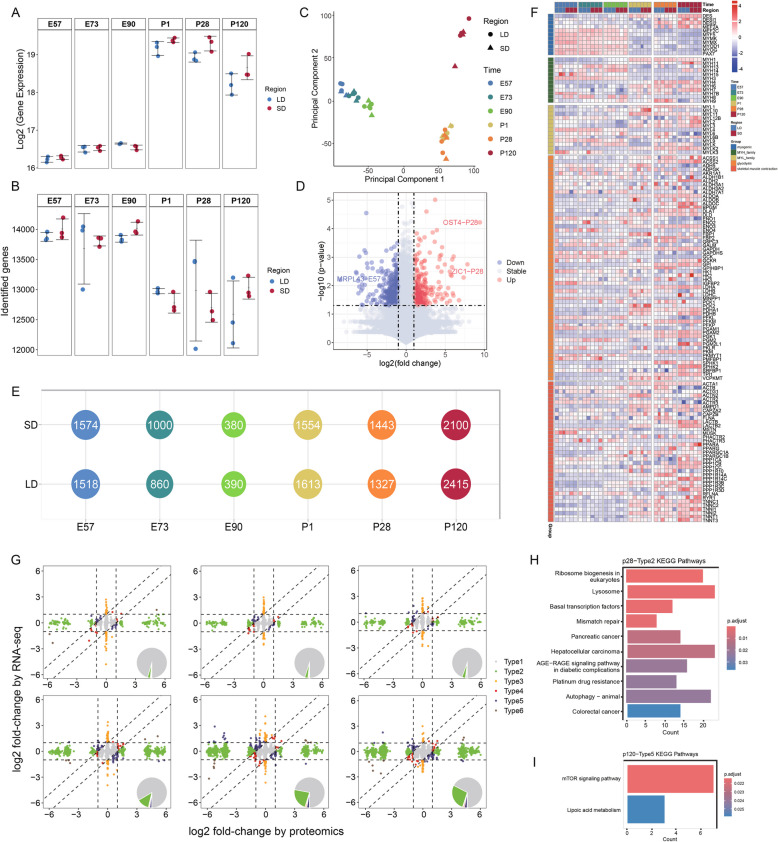
RNA-seq analysis of skeletal muscles and integrated proteome-transcriptome profiling. **A**, **B** The total gene expression levels (**A**) and the number of identified genes (**B**) across the two muscle regions at six developmental stages, with three biological replicates per stage. The average number of identified proteins per stage is also presented. The dots represent individual sample values, the horizontal lines indicate the mean values, and the error bars denote the standard deviations. **C** PCA illustrating the distribution of all samples based on gene expression, with colors indicating different stage groups. Circles denote samples from the LD, and triangles represent samples from the SD. **D** Volcano plot comparing LD and SD, with genes exhibiting Log2|FC|> 7.5 annotated by gene name and corresponding period. **E** The number of DEGs identified in distinct regions and stages is presented. DEGs for each region were determined by comparison with other muscle regions at the corresponding developmental stage. **F** Heatmap depicting the expression patterns of key genes categorized into five groups: myogenic genes, MYH family, MYL family, glycolysis-related genes, and skeletal muscle contraction-related genes (*n* = 3 per group). **G** Scatter plot depicting FC between prenatal and postnatal samples for RNA-seq and proteomics across the skeletal muscle regions (LD, SD). Genes are classified into six categories based on RNA and protein level FC, as described in the "Methods" section. Each category is represented by a distinct color, and pie charts in the inset display the relative percentages of each gene category. Source data are provided as a Source Data file. **H** Top 10 KEGG biological processes enriched in type 2 genes at P28. **I** KEGG biological processes associated with type 5 genes at P120

The RNA profiles of the 36 samples were quantified through transcriptomic analysis. Protein abundance was expressed as TPM (transcripts per million) and was transformed log2 before differential expression analysis (Fig. 5A and Supplementary Table 4). On average, 13,354 genes were identified in each sample (ranging from 12,015 to 14,196) (Fig. 5B and Supplementary Table 5). DEGs were determined using the Student’s t-test with FDR correction. The number of DEGs across the six stages in different muscle regions is illustrated in Fig. 5E. However, transcript profiling across different skeletal muscle regions did not show clear separation along developmental stages in the PCA plot, just as protein PCA results (Fig. 5C). To investigate the expression differences between the two skeletal muscle regions, volcano plots of DEGs between the two regions were generated, revealing that at P28, the LD region exhibited significant upregulation of genes such as OST4 and ZIC1, while at E57, the SD region showed markedly higher expression of the MRPL4 gene compared to LD (Fig. 5D). Similarly, a heatmap of key genes was generated using the same methodology as previously described (Fig. 5F). The heatmap reveals distinct expression patterns, such as the elevated expression of myogenesis-related genes during the embryonic stage, while genes associated with muscle structure, function, and glycolysis in muscle tissue exhibit higher expression postnatally. Overall, the transcriptomic and proteomics data show a high degree of consistency.

To investigate the discrepancies between RNA and protein expression levels and to explore post-transcriptional regulatory mechanisms, we compared proteomic and transcriptomic alterations across the six developmental stages between the two muscle regions. Based on FC in both RNA and protein expression, genes were classified into six categories (Fig. 5G and Supplementary Table 6). Type 1 genes were characterized by minimal alterations in both RNA and protein levels (|FC|< 2). Type 2 and Type 3 genes exhibited substantial discrepancies between RNA and protein levels, with Type 2 genes displaying significant changes at the protein level, and Type 3 genes exhibiting significant changes at the RNA level. Type 4 genes showed consistent directional changes with fold changes > 2 at either the RNA or protein level. Type 5 genes demonstrated an inverse directionality between RNA and protein levels. Type 6 genes exhibited consistent directional changes with fold changes > 2 at both the RNA and protein levels. Types 1–3 accounted for more than 90% of all genes. The proportion of Type 2–6 genes during fetal stages remained below 10%. Notably, starting from P1, the proportion of Type 2 genes exceeded 10% (12.80%) and progressively increased over time (P28, 23.04%; P120, 27.65%), while Type 3 genes showed minimal change. This indicates significant discrepancies between RNA and protein levels. A similar pattern was observed for Type 5 genes, with their proportion consistently exceeding 2% postnatally, while it remained below 1% during fetal stages (P1, 2%; P28, 2.68%; P120, 2.78%). Since the proportion of Type 2 genes increased significantly after P28, exceeding 20%, we further explored the enrichment of Type 2 genes at this stage. At P28, Type 2 genes were predominantly enriched in transcription-related processes, such as ribosome biogenesis in eukaryotes, lysosome function, and basal transcription factors (Fig. 5H). In the GSEA enrichment analysis of type 2 genes at P28, a significant association with fatty acid metabolism was observed (Fig. S7E). In addition, at P120, Type 5 genes exhibited the highest proportion among all time points and were primarily enriched in the mTOR signaling pathway and lipoic acid metabolism (Fig. 5I). These findings highlight the crucial role of proteomics research in muscle development and complement previous transcriptomic studies.

### Cross-species comparison of transcriptomic profiles and functional enrichment at weaning points

Since muscle fiber type transformation is particularly active around P28 in pigs, we initially intended to conduct a cross-species comparison of muscle differentiation during this key developmental period. However, we found a lack of relevant transcriptomic data for humans and mice during comparable stages, making direct comparisons unfeasible. Notably, P28 in pigs not only Marks a critical phase of muscle differentiation but also coincides with the weaning period. Moreover, transcriptomic data for the weaning stage are relatively abundant across species. Therefore, we used the weaning time point as a reference for cross-species analysis. To investigate whether similar gene expression patterns also exist in other species, we analyzed the transcriptome data of pigs, humans and mice during the weaning period. For humans and mice, we respectively selected the developmental time points corresponding to the weaning period, which were 7 months and 20 days. All the selected stages represent the period after birth. There are a total of 1,869 homologous genes in the three species. These homologous genes were determined through pairwise comparisons of single-copy genes among pigs, humans and mice (Supplementary Table 7).

**Fig. 6:**
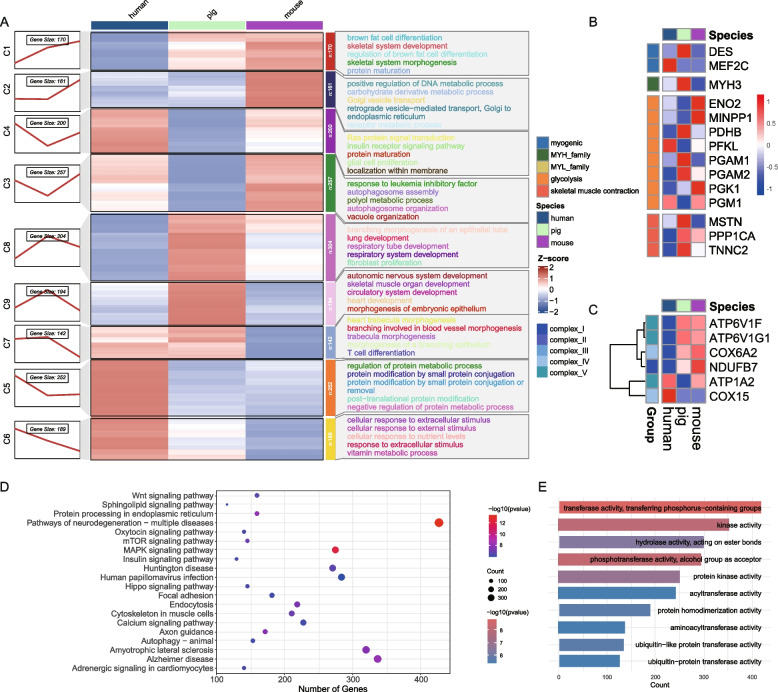
Comparative transcriptomic profiling of human, pig, cattle, and mouse skeletal muscles. **A** RNA-seq analysis reveals that the expression patterns across the four species can be categorized into eight distinct clusters based on their expression dynamics. The temporal trends for each cluster are depicted in the line chart in the left panel. Z-scores for each species are shown in the middle panel. The right panel presents KEGG enrichment results for the identified clusters. **B** Heatmap depicting the expression patterns of key genes categorized into five groups: myogenic genes, MYH family, MYL family, glycolysis-related genes, and skeletal muscle contraction-related genes across four species. **C** Heatmap depicting differentially abundant proteins associated with respiratory complexes in skeletal muscle across four species. **D** The top 20 KEGG biological processes significantly enriched in non-homologous genes. **E** The top 5 GO biological processes enriched in non-homologous genes

Based on the expression profiles of these homologous genes, we observed that compared with the other two species, the expression levels of pig-specific clusters C8 and C9 were relatively high, while the expression levels of clusters C3 and C4 were relatively low (Fig. 6A). Interestingly, the cluster C8 gene, which is highly expressed in pigs, is significantly enriched in lung development-related pathways, such as lung development, respiratory tube development, and respiratory system development. In contrast, the pig-specific high-expression genes in cluster C9 were enriched in pathways associated with skeletal muscle organ development and other organogenesis processes, including circulatory system development and heart development. On the other hand, pig-specific low-expression clusters showed distinct patterns: C3 genes were enriched in autophagy-related pathways such as autophagosome assembly and autophagosome organization, while C4 genes were associated with key signaling pathways, including Ras protein signal transduction and insulin receptor signaling.

We further identified and visualized key muscle-related orthologous genes (Fig. 6B) as well as mitochondrial complex-related genes (Fig. 6C). Notably, the majority of contraction-related genes exhibited higher expression in mice at their weaning stage (P28) compared to pigs and humans (Fig. 6B). In the mitochondrial complex heatmap (Fig. 6C), orthologous genes were identified primarily in Complexes I, IV, and V. Interestingly, pigs and mice displayed a coordinated expression pattern for these mitochondrial genes, whereas humans exhibited an opposing trend. In the lower region of the heatmap, for instance, these genes showed elevated expression in humans but lower levels in pigs and mice. This divergence may be attributed to species-specific differences in gestational length: humans have a longer gestation period (~ 10 months) (Polin et al. 2017) and a correspondingly delayed weaning period. These developmental differences may partially explain the observed discrepancies in gene expression profiles among the species.

Additionally, we performed KEGG and GO enrichment analyses on non-orthologous genes in pigs. KEGG analysis revealed that pathways such as the Wnt signaling pathway, Oxytocin signaling pathway, and Protein processing in the endoplasmic reticulum (Fig. 6D). Enrichment of GO further indicated significant involvement of transferase activity, kinase activity, and hydrolase activity (Fig. 6E).

Taken together, our findings indicate that pigs exhibit elevated expression of genes related to skeletal muscle and lung development during weaning. This may reflect the biological adaptations related to their earlier weaning schedule compared to humans and mice.

## Discussion

The difference of skeletal muscle fiber types is one of the key factors affecting the meat quality of livestock and poultry. Therefore, elucidation of the key genes controlling the regulation of muscle fiber type and their underlying mechanisms is crucial to improve meat quality traits. Skeletal muscle exhibits remarkable plasticity, which is closely related to the regulation of mitochondrial activity, including ATP production and calcium uptake capacity (Dong and Tsai 2023). Oxidized fiber types (types I and IIa) show higher mitochondrial content compared with glycolytic fibers (type IIb) (Finck 2006; Scarpulla 2008). Compared with glycolytic fibers, oxidized fibers are characterized by increased mitochondrial bulk density and enhanced expression of mitochondrial complex proteins (Herbst et al. 2017; Frankish et al. 2021). These unique features highlight the critical role of mitochondria in determining the metabolic properties of different muscle fiber types. Mitochondrial energy metabolism is enhanced during postnatal development, with major effects on muscle fiber differentiation and function (Kim et al. 2024). Consistent with these observations, our study identified significant differences in skeletal muscle fiber types during porcine P1-P28 development. Specifically, we identified the most pronounced differences in protein abundance changes and their corresponding enriched signaling pathways during this timeframe compared to other developmental stages (Fig. 2C, D, Fig. S1B, C). These observations align with previous research, which indicated that oxidative and glycolytic fibers in pigs can be distinguished from 21 to 28 days postnatal (Davies 1972). Moreover, our heat map analysis of mitochondrial complex-related proteins revealed that differences between two skeletal muscle types began to emerge at P28 and reached their peak at P120 (Fig. 2E). This highlights the P28 stage as a critical window for skeletal muscle fiber conversion. Our findings are further supported by a nine-quadrant diagram integrating transcriptomic and proteomic data, which reveals a significant increase in Type 2 genes—characterized by substantial differential protein expression but minimal differential gene expression—between the two skeletal muscles beginning at P28 (Fig. 5G). It is worth noting that these genes are mainly enriched in ribosomal biogenesis (Fig. 5H), which is consistent with recent studies (Moreno-Justicia et al. 2025), indicating that ribosomal heterogeneity in skeletal muscle fibers contributes to variations that are not related to slow or fast fiber types. Furthermore, GSEA of the P28 Type 2 gene set revealed significant enrichment in the fatty acid metabolism pathway (Fig. S7E). This indicates that genes showing differential protein abundance but not transcriptomic change between LD and SD muscles are associated with this metabolic process. This finding is consistent with previous studies that showed that the activities of enzymes involved in fatty acid metabolism were higher in slow muscle fibers (Lee et al. 2022). These results highlight the functional relevance of fatty acid metabolism in defining the different energy spectra of different muscle fiber types. Overall, our results highlight the critical role of P28 in myofiber transformation, based on changes in mitochondrial function, metabolic pathway engagement, and protein abundance.

Proteomics represents a sophisticated analytical methodology in scientific investigation with multifaceted applications, particularly in agricultural animal research. It serves as an instrumental approach for elucidating biological processes that influence meat quality traits in livestock. Proteomic analyses have been effectively deployed to examine meat quality characteristics in porcine subjects (Wei et al. 2022; Yu et al. 2023) and developmental processes (Chen et al. 2024). Similarly, our previous iTRAQ-based proteomic study (Zhang et al. 2016) provided insights into muscle fiber development and breed-specific characteristics. However, most studies have focused on specific developmental stages or muscle types, leaving a gap in the understanding of muscle fiber differentiation. To bridge this gap, we analyzed six prenatal and postnatal stages of two skeletal muscle types in BM pigs. Using advanced nDIA strategy proteomics, we achieved higher resolution, improved protein quantification, and broader proteome coverage (Guzman et al. 2024). This approach allowed a detailed analysis of protein expression patterns, particularly for fast- and slow-twitch muscle fibers. These findings enhance knowledge of muscle development, offering strategies to improve meat quality. Despite these scientific advances, BM pigs face several inherent disadvantages compared to commercial breeds such as Landrace, including a low percentage of lean meat, high fat content, slow growth rate, and poor feed conversion efficiency (Zhu et al. 2020a, b; Wang et al. 2023). These traits significantly limit their commercial viability. However, the underlying mechanisms of muscle development in BM pigs remain largely unexplored. Given that skeletal muscle traits are closely related to meat quality and growth performance, a better understanding of muscle development in BM could provide new strategies for improving these traits. Therefore, employing the nDIA proteomics technique in combination with transcriptomic data to further investigate the muscle development mechanisms in BM pigs is of both scientific and practical importance. This integrated approach not only deepens our understanding of muscle fiber differentiation but also provides valuable insights for improving the commercial traits of BM pigs.

Through structural domain enrichment and tendency analysis of differential proteins, NEK3 has been identified as a critical protein-coding gene with a significant regulatory role in muscle fiber type differentiation. Previous studies have shown that NEK3 regulates organelle dynamics and cytoskeletal structure (Polgár et al. 2003). Furthermore, experimental evidence from a study indicates that NEK3 regulates cytoskeletal recombination and promotes cell migration and invasion (Miller et al. 2007). These findings collectively emphasize the potential role of NEK3 in the structural and metabolic remodeling of skeletal muscle fibers. Furthermore, genetic associations analyzed by GWAS (Genome-wide Association Studies) revealed that NEK3 was associated with weight-related genetic variations (Zhong et al. 2024). Given that skeletal muscle is the most abundant tissue of mammals, comprising 40–50% of the total body mass (Choi and Kim 2009), this association suggests that NEK3 may indirectly influence muscle fiber type transitions to modulate systemic skeletal muscle development. Our research further characterized the dynamic and muscle-specific expression profile of NEK3, highlighting its stage-dependent regulatory function. Interestingly, research on the metabolic function of NEK3 is still scarce. To address this knowledge gap, our goal is to further explore the role of this gene in skeletal muscle. Immunofluorescence staining in C2C12 cells and embryonic muscle progenitors indicated that NEK3 inhibits myogenesis (Fig. S7B, Fig. 4I), and western blot analysis of NEK3-overexpressing cells demonstrated a suppression of fast-twitch fiber differentiation (Fig. S7D, Fig. 4J). Notably, our proteomic data showed that NEK3 emerged as a DAP between LD and SD at P1, with a striking log2|FC| exceeding 10. However, this regional difference diminished in subsequent stages, and NEK3 was no longer identified as a DAP at other time points. In contrast, transcriptomic analysis revealed that the expression levels of NEK3 were consistent in both regions and it had never been classified as a region-specific DEG. These findings suggest that NEK3 may be regulated after transcription, highlighting the significance of multi-omics approaches in revealing complex regulatory mechanisms beyond gene expression. These findings suggest that NEK3 dynamically regulates muscle development to meet the different physiological needs of various muscle types. In conclusion, our research results indicate that NEK3 is a key regulatory factor for the type conversion of skeletal muscle fibers. In addition, NCOA2 exhibits a distinct temporal expression pattern in the LD and SD muscles. Although its overall expression tends to decline in both muscle types, the onset of this decrease differs significantly. In the LD muscle, NCOA2 expression begins to decline at E73 and reaches zero by P28 (Fig. 4B). In contrast, in SD muscle, the decline started later, at E90, and did not reach zero until P120 (Fig. 4D). This temporal difference may be closely related to the specific role of NCOA2 in skeletal muscle development. Previous studies have identified NCOA2 as a key regulator of skeletal muscle oxidative metabolic homeostasis (Duteil et al. 2010), which may act to prevent the transition of slow to fast muscle fibers. Thus, the different temporal patterns of NCOA2 expression observed in LD and SD may reflect its role in fine-tuning myofiber type specification during development. Given that the majority of fetal muscle fibers in pigs exhibit slow-twitch characteristics before birth (Joo et al. 2013), NCOA2 is highly expressed in both muscle types during early developmental stages. As development progresses, the LD undergoes differentiation toward a fast-twitch fiber-dominant phenotype. During the critical postnatal transition from P1 to P28, NCOA2 expression declines rapidly. This downregulation may relieve its inhibitory effect on the transition of slow to fast muscle fibers, thereby promoting fast fiber specification. In contrast, the SD retains slow-twitch characteristics for a more extended period. As a result, the reduction in NCOA2 expression occurs later and does not approach near-complete suppression until the pig reaches full maturity. Overall, these findings suggest that NCOA2 may act as a molecular brake on the slow-to-fast muscle fiber transition, with its downregulation serving as a critical switch to enable fiber-type remodeling during development.

Weaning is a critical stage in the pig production system as it has a significant impact on the economic efficiency of pig operations (Tang et al. 2022). Contemporary intensive farming methodologies frequently incorporate early weaning protocols to optimize sow reproductive performance, thereby increasing annual litter production, enhancing breeding facility utilization, and maximizing economic returns for production enterprises (Campbell et al. 2013). Given these advantages, understanding species-specific gene expression in pigs during the weaning period is of paramount importance. In our cross-species analysis, we observed that genes associated with both lung and skeletal muscle development were significantly enriched at P28 (Fig. 6A), a finding consistent with previous research. Notably, an in vitro study has shown that mechanical stretching promotes the differentiation of fetal type II alveolar epithelial cells via growth factor signaling, and that this process depends on mechanical stimuli generated by the respiratory musculature (Mark et al. 2012). This highlights a developmental interdependence between the lungs and skeletal muscles. It is plausible that the enrichment of both pathways during this period reflects this physiological relationship: at P28, skeletal muscles are undergoing a critical phase of muscle fiber type conversion, a process that enhances their contractile capacity. The mechanical force produced by these developing muscles may, in turn, support proper lung development, thereby triggering the expression of related developmental pathways. In summary, these findings suggest a coordinated developmental program between skeletal muscle and lung tissues during the weaning period in pigs. Understanding this interplay not only enhances our biological insight into postnatal development but may also inform strategies to optimize health and productivity during this economically vital phase of pig farming.

## Supplementary Information

Supplementary Material 1.

Supplementary Material 2.

Supplementary Material 3.

Supplementary Material 4.

Supplementary Material 5.

Supplementary Material 6.

Supplementary Material 7.

Supplementary Material 8.

Supplementary Material 9.

## Data Availability

RNA-seq data of skeletal muscle for human, and mouse were obtained from GSE257558, and GSE167665, respectively. Porcine muscle RNA-seq data are stored in the GSA database with the number CRA023919. The data of this research will be made available on request.

## Abbreviations

LD: Longissimus dorsi
SD: Semitendinosus
NEK3: Never In Mitosis Gene A-Related Kinase 3
S_TKc: Serine/Threonine protein kinases, catalytic domain
nDIA/MS–MS: Non-targeted data-independent acquisition and tandem mass spectrometry
ATP2B1: ATPase Plasma Membrane Ca2+ Transporting 1
UQCC4: Ubiquinol-Cytochrome C Reductase Complex Assembly Factor 4
COX15: Cytochrome C Oxidase Assembly Homolog COX15
ATP1B4: ATPase Na+/K+ Transporting Family Member Beta 4
SDHB: Succinate Dehydrogenase Complex Iron Sulfur Subunit B
NDUFB6: NADH Ubiquinone Oxidoreductase Subunit B6
NDUFS7: NADH Dehydrogenase [Ubiquinone] Iron-Sulfur Protein 7,
UBQ: Ubiquitin homologues
UBA: Ubiquitin associated domain
HLH: Helix loop helix domain
NCOA2: Nuclear Receptor Coactivator 2
MEF2A: Myocyte Enhancer Factor 2A
MYOD: Myogenic Differentiation 1
MYH4: Myosin Heavy Chain 4
MYH7: Myosin Heavy Chain 7
PGC-1α: Peroxisome Proliferator-Activated Receptor Gamma Coactivator 1-Alpha
MYHC: Myosin Heavy Chain 6
ST4: Crystallin Beta-Gamma Domain Containing 1
ZIC1: Zic Family Member 1
MRPL4: Mitochondrial Ribosomal Protein L4
GWAS: Genome-wide association studies
