# Supplementary material for: Integrated proteomics and transcriptomics analysis of dynamic changes in muscle fiber types in different regions of porcine skeletal muscle
Source: Adv Biotechnol (Singap). 2025 Sep 24;3(4):29. doi: 10.1007/s44307-025-00080-w (PMC12460891; doi:10.1007/s44307-025-00080-w)
Supplement: Supplementary file 1 — Supplementary Material 1. [file 44307_2025_80_MOESM1_ESM.docx]

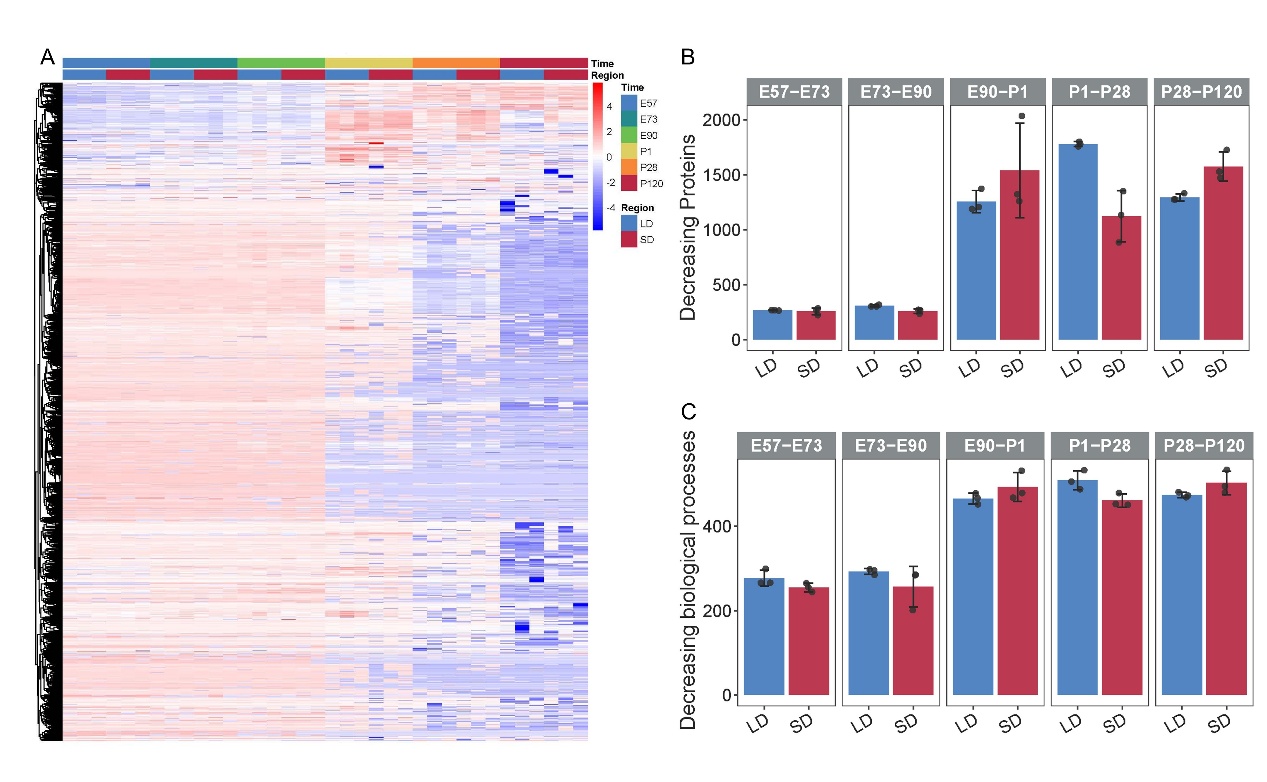


**Figure S1** A. Heatmap illustrating whole proteins abundance, clustered by k-means. B, C. The number of decreasing identified proteins (B) and decreasing enriched biological processes (C) in the LD and SD regions when comparing adjacent stages.


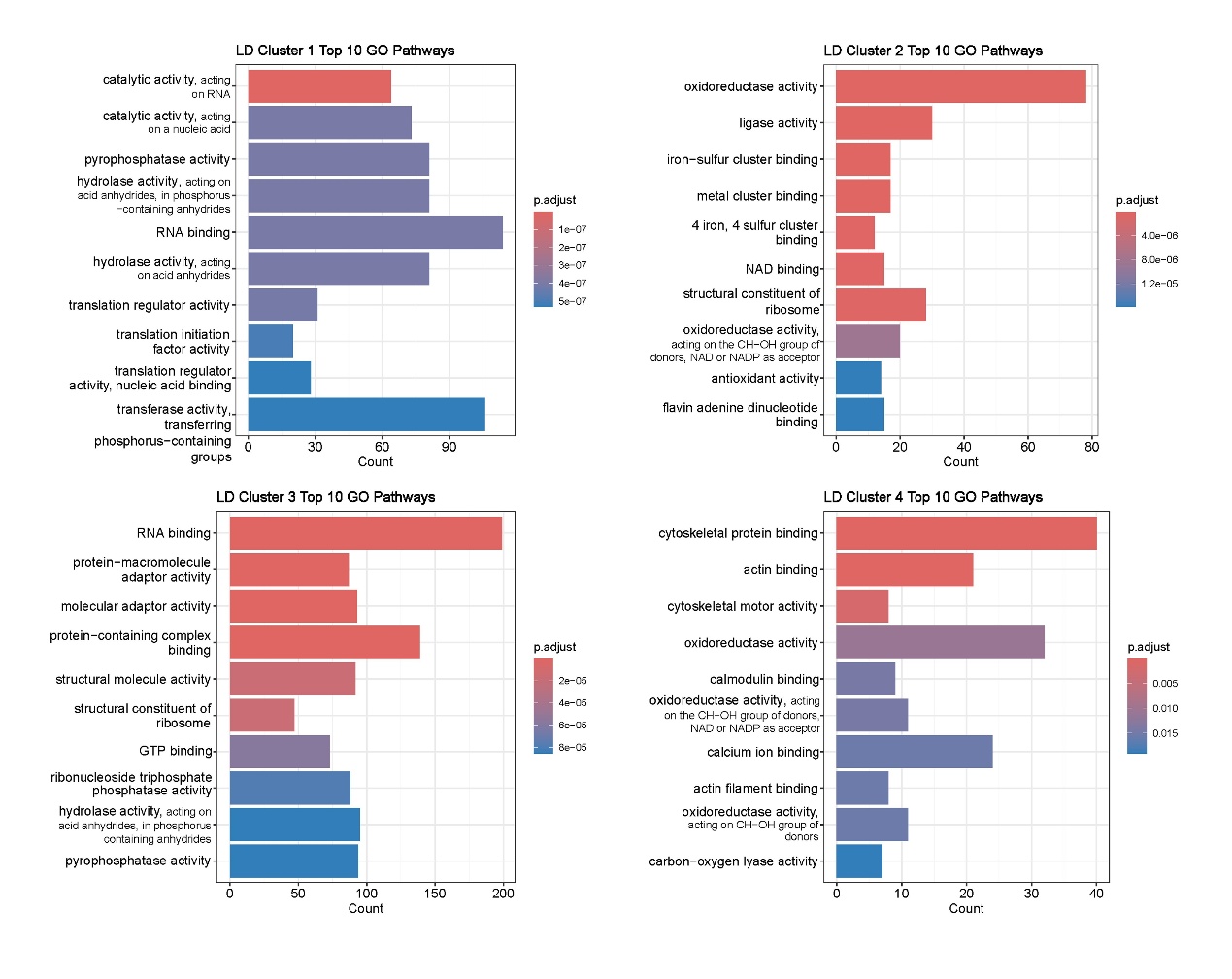


**Figure S2** Top 10 GO significantly enriched pathways of LD across four distinct tendencies.


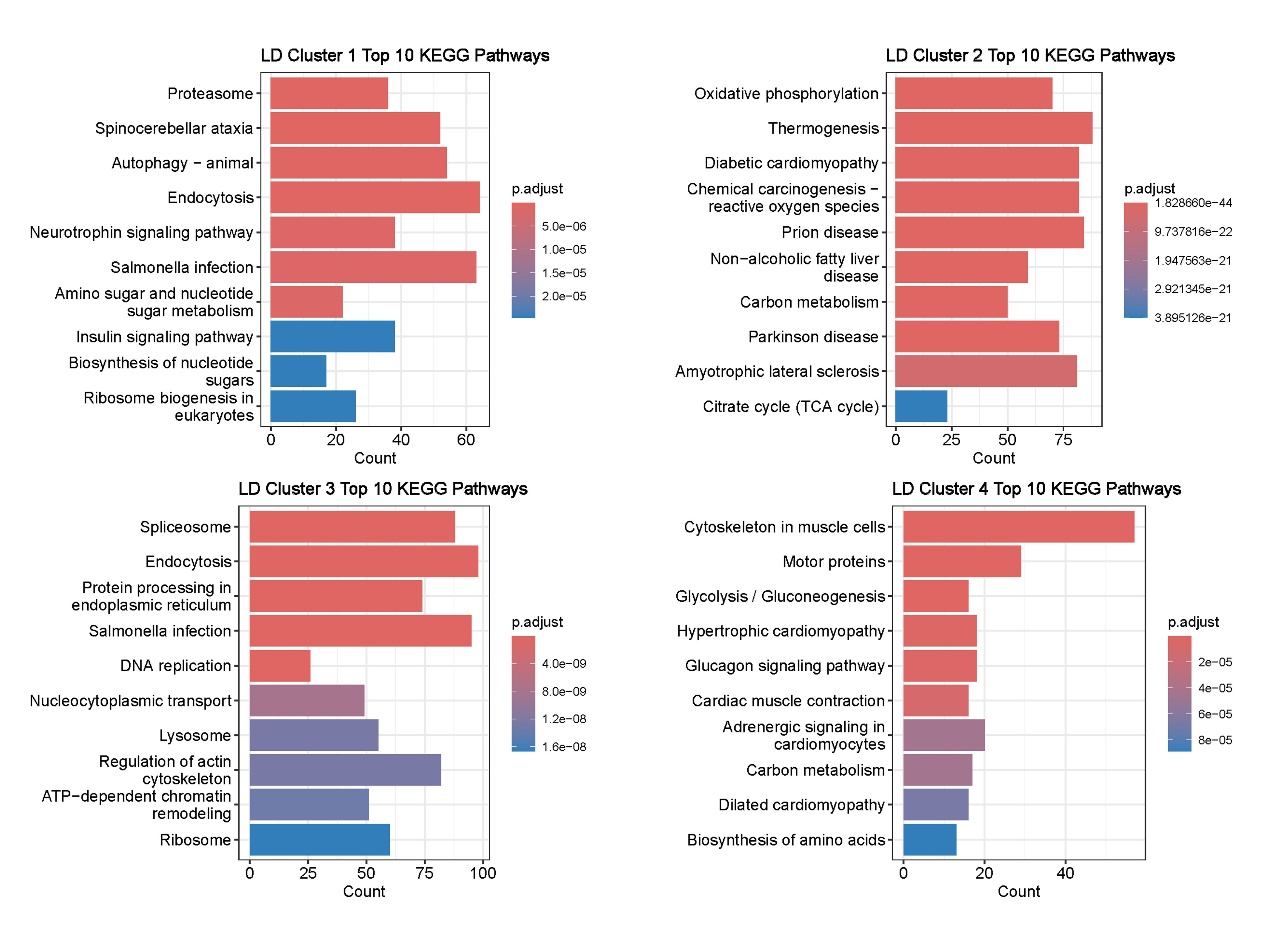


**Figure S3** Top 10 KEGG significantly enriched pathways of LD across four distinct tendencies.


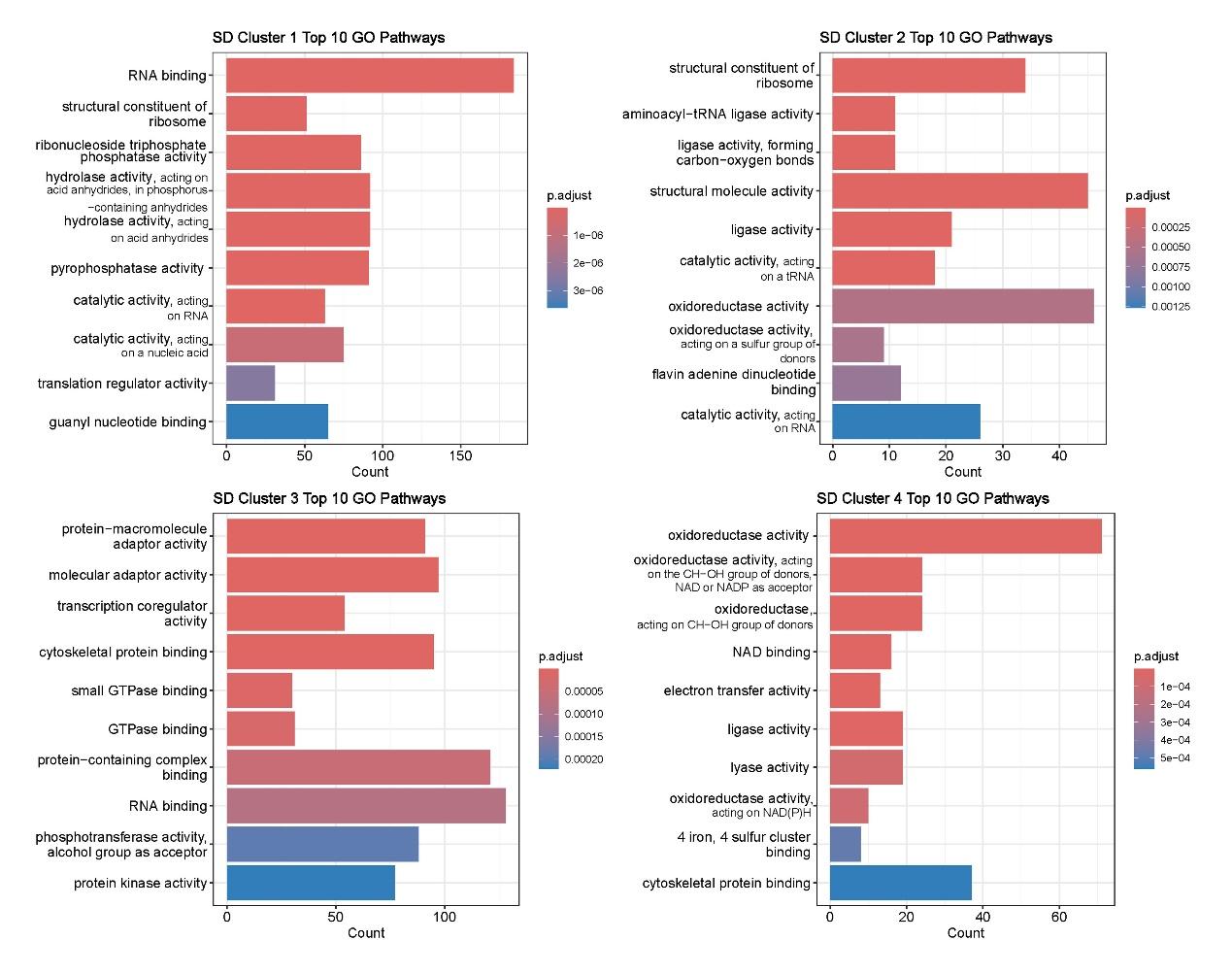


**Figure S4** Top 10 GO significantly enriched pathways of SD across four distinct tendencies.


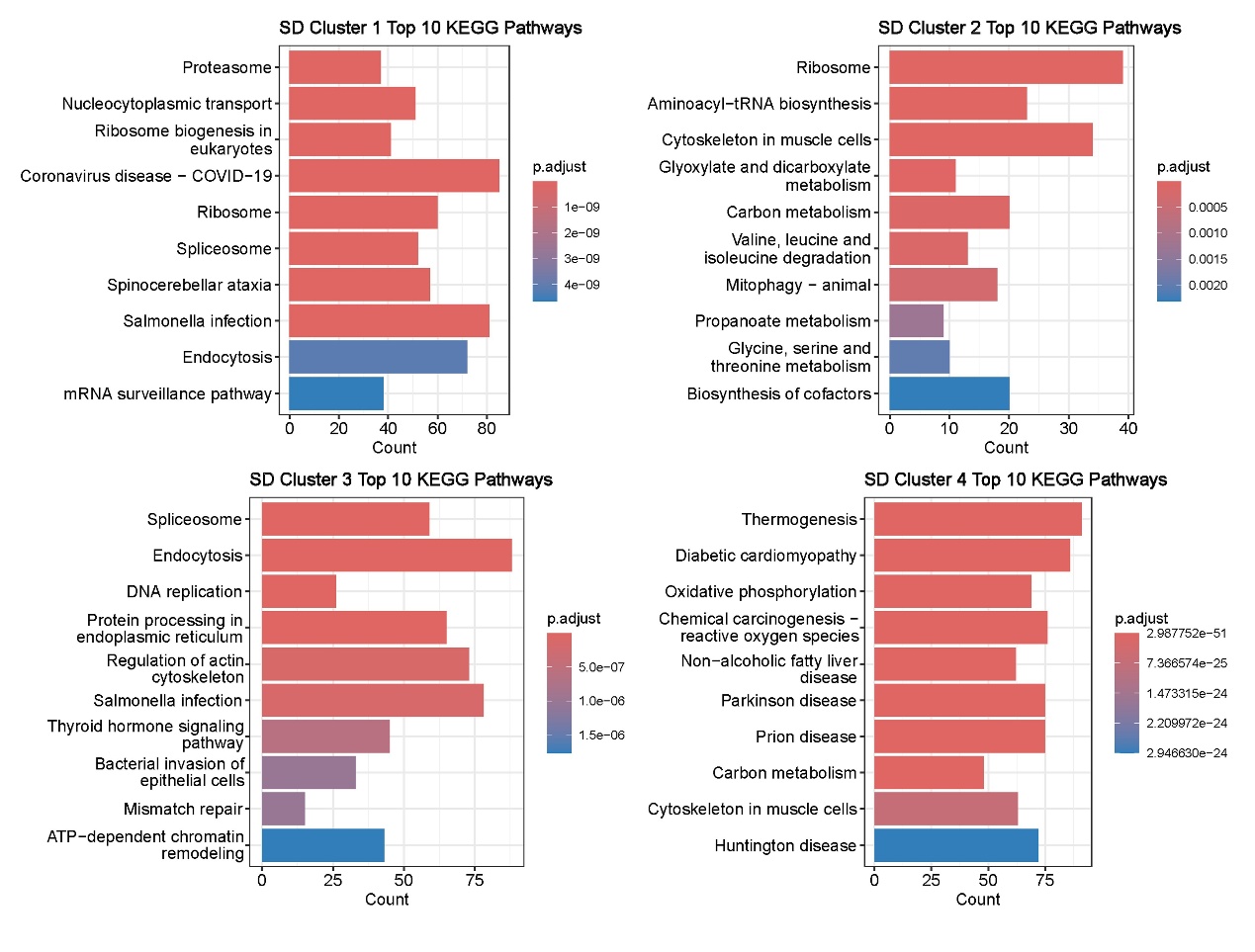


**Figure S5** Top 10 KEGG significantly enriched pathways of SD across four distinct tendencies.


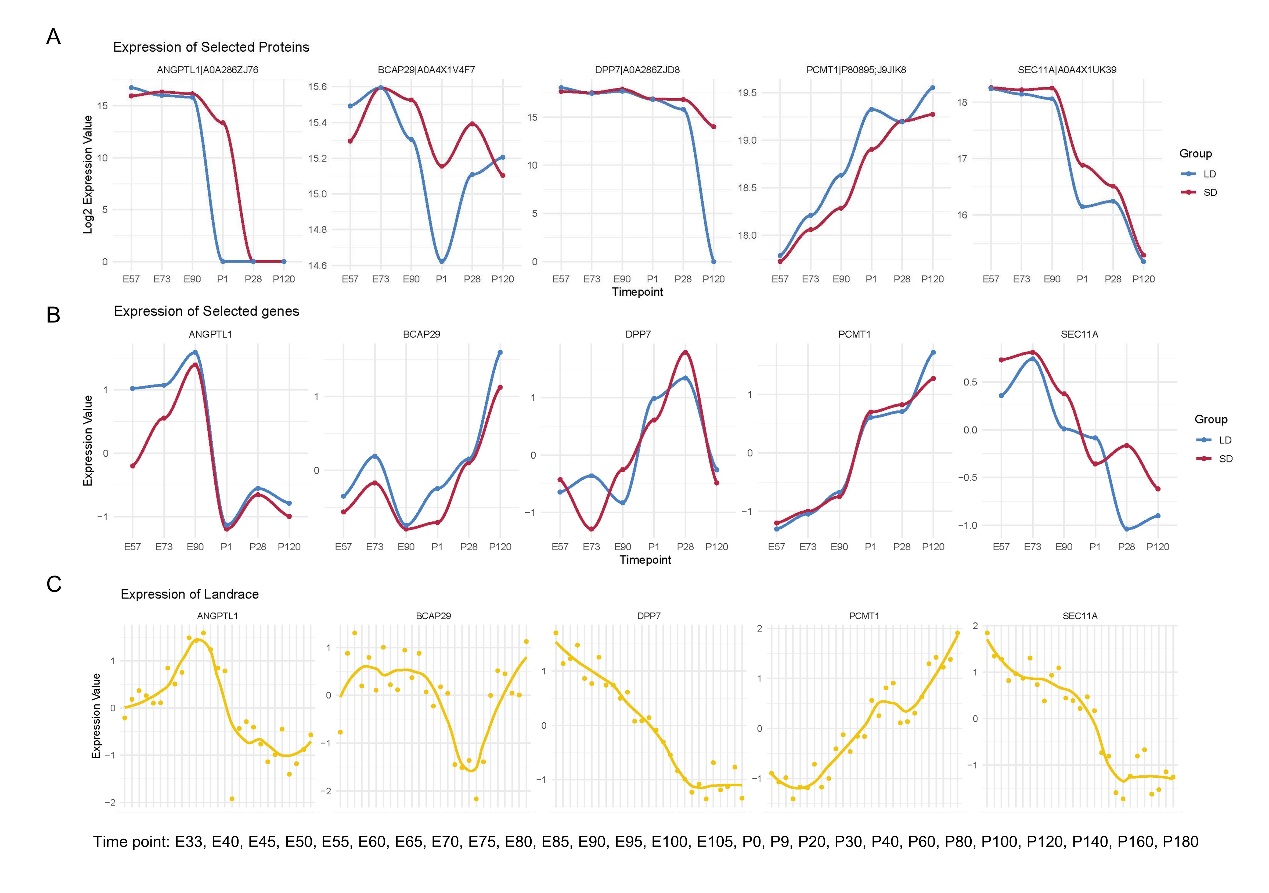


**Figure S6** A. Line plots illustrating protein abundance levels for selected genes from the proteomics data. B. Line plots depicting gene expression levels for selected genes from the transcriptome data. C. Line plots showing gene expression levels for selected genes from the previously published Landrace longissimus dorsi transcriptome data.


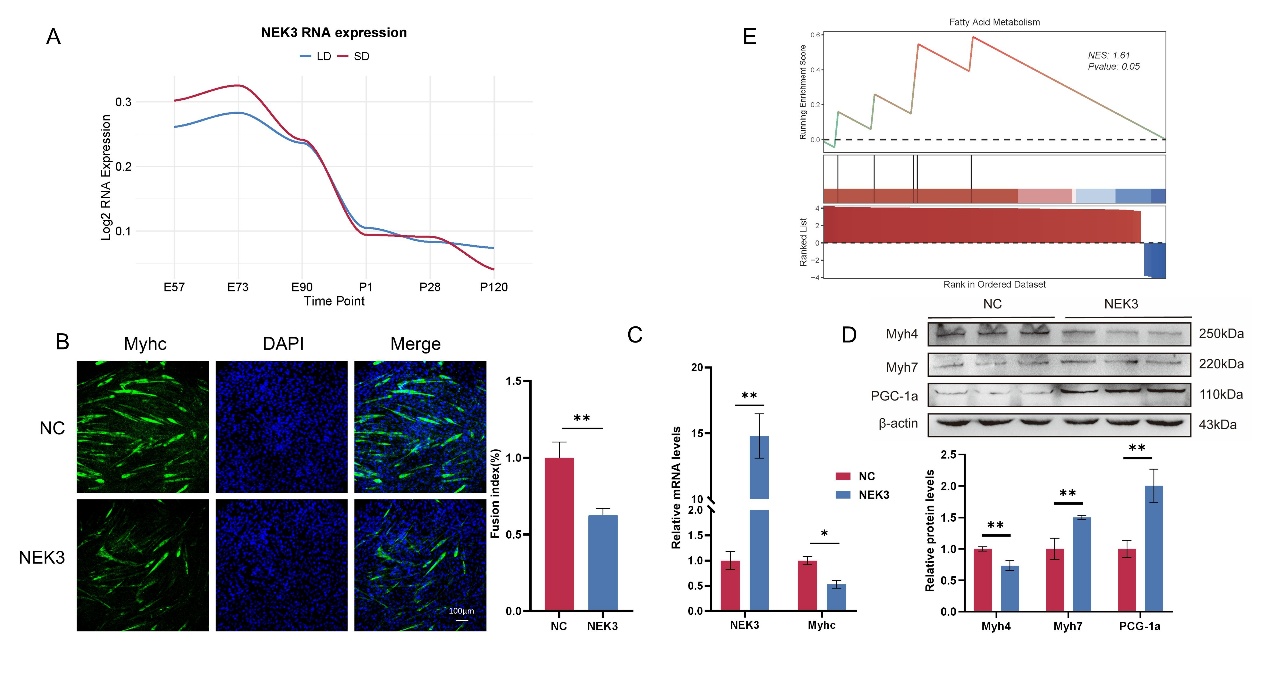


**Figure S7** A. RNA expression patterns of NEK3 in LD and SD. B. MyHC immunofluorescence staining after four days of myogenic induction. Upper panel: MyHC (green); DAPI (blue). Scale bar: 100 μm (n = 3). Lower panel: Fusion index of cells. C. Quantification of NEK3 and MyHC mRNA levels in NC C2C12 cells and NEK3-overexpressing C2C12 cells (n = 3). D. Western blot analysis of Myh4, Myh7, and PGC-1α expression in C2C12 cells and NEK3-overexpressing cells (n = 3). *P < 0.05, **P < 0.01, **P < 0.001 (Student’s t-test). E. GSEA enrichment analysis of the Type 2 gene set at P28.
